# An Angle-Dependent Bias Compensation Method for Hemispherical Resonator Gyro Inertial Navigation Systems

**DOI:** 10.3390/s25216639

**Published:** 2025-10-29

**Authors:** Chao Liu, Qixin Lou, Ding Li, Huiping Li, Tian Lan, Yutao Wu, Hongjie Meng, Jingyu Li, Tao Xia, Xudong Yu

**Affiliations:** 1 College of Advanced Interdisciplinary Studies, National University of Defense Technology, Changsha 410073, China; liuchao_1@nudt.edu.cn (C.L.); louqixin22@nudt.edu.cn (Q.L.); liding97@nudt.edu.cn (D.L.); lihuiping23@nudt.edu.cn (H.L.); lantian23@nudt.edu.cn (T.L.); wuyutao24@nudt.edu.cn (Y.W.); mhj.nudt@nudt.edu.cn (H.M.); lijingyu13c@nudt.edu.cn (J.L.); xiat06@163.com (T.X.); 2 Nanhu Laser Laboratory, National University of Defense Technology, Changsha 410073, China

**Keywords:** hemispherical resonator gyro (HRG), inertial navigation system (INS), system-level calibration, Kalman filter, whole-angle (WA) mode

## Abstract

In the whole-angle mode of a hemispherical resonator gyro (HRG), the external input rotation angle is obtained by detecting the standing-wave rotation angle through electrodes. Due to this operational principle and manufacturing constraints of HRGs, the gyro output in an HRG inertial navigation system exhibits angle-dependent errors that are highly sensitive to temperature variations. To address this issue, this paper proposes a system-level calibration scheme to characterize and compensate for these correlated errors. Angle-dependent bias models were established through multi-temperature point experiments. A Kalman filter was subsequently designed, and a calibration path satisfying observability requirements was developed. System-level calibration experiments were conducted to determine and compensate for the identified errors. Finally, navigation experiments demonstrated the effectiveness of the proposed method, showing that the navigation accuracy of the HRG inertial navigation system was improved by up to 94.35%.

## 1. Introduction

Inertial Navigation System (INS) is a self-contained navigation system that relies solely on its internal sensors to perform navigation. The core component of this system is the Inertial Measurement Unit (IMU), typically composed of three gyroscopes and three accelerometers. The gyroscopes measure angular motion, while the accelerometers measure specific force. Based on these measurements, the system can calculate the vehicle’s motion parameters including attitude, velocity, and position. The Strapdown Inertial Navigation System (SINS), where the inertial navigation system is directly mounted on the vehicle, represents the most prevalent type of INS in practical applications today.

As a high-precision core sensor in inertial navigation systems, the gyroscope’s performance accuracy and error control capability directly determine the precision of the entire system. The Hemispherical Resonator Gyro (HRG) is a high-precision, highly reliable, and miniaturized angular rate sensor, acclaimed as a “disruptive technology.”

Research on hemispherical resonator gyroscopes can be traced back to 1890, when G.H. Bryan discovered the inertial properties of elastic waves [1,2]. In 1965, Dr. David Lynch at the Delco Research Center developed the first theoretical model based on G.H. Bryan’s theory using the Lagrangian method. Building on this foundation, he designed, manufactured, and tested two experimental HRG prototypes [2]. The first experimental results on HRG were obtained and published by D.D. Lynch in 1984. In 2012, Meyer and Rozelle created a novel mHRG system that not only reduced its size but also achieved a bias as low as 0.00035°/h through innovative self-calibration technology [3]. In 2019, Delhaye from Safran introduced the latest HRG research outcome—the SkyNaute inertial system—a disruptive navigation system for commercial aircraft characterized by its miniaturization, light weight, low power consumption, high reliability, and low cost [4]. Chikovani et al. proposed a method to significantly reduce gyro bias error by adjusting the standing-wave angle [5]. In the most recent research progress, Chikovani further introduced a new differential operating mode for HRG, which automatically compensates for frequency mismatch, suppresses external disturbances, and allows free switching among three operating modes according to different application scenarios [6].

The hemispherical resonator gyro measures external angular rate or angle by detecting the precession of the standing wave in the resonator [7]. Under external excitation, the resonator generates a four-node standing-wave pattern. When an external rotational angular rate is applied, the Coriolis force causes the standing-wave pattern to precess relative to the housing [8,9,10,11]. The precession angle θ is proportional to the external rotation angle. The base electrodes measure this precession angle to determine the external rotation angle.

The standing wave in the resonator typically exhibits a four-antinode pattern. When the external casing rotates about its central axis, the relationship between the precession angle variation φ(t) of the standing wave and the input angular rate Ω is given by [12]:

(1) $$\varphi (t)=-\frac{2}{k^{2}+1}\int_{0}^{t}Ω(\tau )d\tau$$

where k = 2 represents the second-order vibrational mode of the resonator.

HRG inertial navigation systems are internationally recognized as the next generation of disruptive, high-precision navigation technologies, offering optimal overall performance in terms of size, weight, power, and cost (SWaP-C) [13,14]. They are suitable for a wide range of applications in navigation, guidance, and control, and serve as core sensing components across various land, marine, aerial, and space platforms.

Due to various error sources in hemispherical resonator gyros (HRGs)—such as asymmetric damping of the resonator, phase error, electrode detection and drive deviations, and nonlinear effects in capacitance sensing—angle-dependent errors arise when the gyro operates in whole-angle mode. Although certain error suppression and compensation techniques have been applied at the device level, harmonic errors have not been entirely eliminated. In HRG-based inertial navigation systems, harmonic bias error and harmonic scale factor error remain present. Therefore, this paper focuses on suppressing and compensating these harmonic errors within HRG inertial navigation systems.

Currently, system-level calibration is the most widely used and effective method for calibrating and compensating errors in inertial navigation systems. Numerous researchers have conducted studies tailored to different types of INS architectures. Camberlein proposed an 18-position calibration path capable of exciting common IMU errors, which is characterized by short calibration time and wide application [15]. Sun derived a minimal calibration path through theoretical analysis [16]. Jiang designed a novel Kalman filter specifically for accelerometer error compensation [17]. In the field of temperature compensation, Weng introduced a modeling method for accelerometer scale factor and bias based on fixed-point heating tests and least squares fitting [18]. Zhang employed statistical tests to determine the optimal polynomial order and established a sparse coefficient temperature regression model along with an RBF neural network compensation model [19]. Yang proposed a cross-validation-based selection criterion for gyro parameter temperature models, applicable under both known and unknown variance conditions [20]. Wei enhanced the robustness of temperature compensation models by incorporating robust estimation theory and investigated the application of least squares support vector machines in compensation [21]. Xu integrated wavelet transform into support vector machine models to effectively suppress pulse noise in temperature compensation [22]. Yang estimated accelerometer temperature model coefficients using a 36-dimensional Kalman filter, incorporating auxiliary measurements from laser gyros [23]. Weng developed a 15-dimensional filter that uses accelerometer-specific force output as observations to achieve online estimation of temperature model coefficients [24]. Lou proposed a system-level calibration method for inertial navigation systems (INS) capable of simultaneously compensating for accelerometer asymmetric errors and second-order temperature-dependent errors in a single calibration process [25]. Tao et al. conducted research on temperature compensation for the Hemispherical Resonator Gyro Inertial Navigation System (HRG INS). Their system-level calibration method requires minimal computational resources and has been shown to effectively improve the calibration accuracy during the system’s start-up phase [26]. However, the error parameters compensated for in their study (such as the biases and scale factors of the gyroscopes and accelerometers) are similar to those of conventional inertial sensors, and they did not explicitly target the modeling of HRG-specific, angle-dependent harmonic error components (e.g., the 4θ term). Furthermore, the study does not specify whether their compensation model is intended for gyro output in force-to-rebalance mode or whole-angle mode. Dong et al. [27] established a nonlinear temperature error compensation model for the hemispherical resonator gyro (HRG) based on the PSO algorithm, incorporating the temperature change rate to enhance compensation capability in varying thermal environments. However, their study specifically addressed the gyro’s output in force-to-rebalance mode and did not account for the angle-dependent error terms unique to the whole-angle mode of the HRG.

System-level calibration has been extensively studied and widely applied in other inertial navigation systems such as ring laser gyros and fiber optic gyros, but these methods are not fully applicable to hemispherical resonator gyro (HRG) inertial navigation systems. Particularly, there is still no effective system-level calibration method for the angle-dependent errors unique to HRGs. This paper models the ADB errors of HRGs and designs a system-level calibration scheme that can calibrate both the ADB errors and their temperature coefficients. This scheme can effectively calibrate various errors in HRG inertial navigation systems, demonstrates good error suppression capability, and thereby significantly improves the navigation accuracy of hemispherical resonator gyro inertial navigation systems.

The main innovations of the proposed method are summarized in the following three aspects:

(1) Introduction of harmonic errors in the HRG-based INS and establishment of the relationship between harmonic bias errors and temperature (frequency).
(2) Development of a novel error model for the hemispherical resonator gyro that incorporates harmonic bias errors and their temperature coefficients.
(3) Design of a new Kalman filter for system-level calibration of the HRG-based INS according to the proposed error model.

This paper is organized as follows: Section 2 analyzes the characteristics of harmonic error versus temperature, the frequency–temperature dependence model, the error modeling approach for the hemispherical resonator gyro, and the formulation of strapdown inertial navigation error equations. Section 3 details the design of a system-level calibration Kalman filter. Section 4 evaluates the system-level calibration scheme and navigation experimental results. Section 5 provides concluding remarks and research findings.

## 2. Error Model

### 2.1. Definition of Reference Frames

(1) The inertial coordinate system (i-frame) has its origin at the center of the Earth, with axes non-rotating relative to the celestial sphere. The three axes are defined as $ox_{i},\;oy_{i},\;oz_{i}$, forming a right-handed coordinate system, where the $oz_{i}$ is aligned with the Earth’s polar axis.
(2) The navigation coordinate system (n-frame) has its origin at the position of the vehicle. Its axes are aligned with the directions of North (N), East (E), and Down (D). Navigation computations are typically performed in the n-frame.
(3) The body coordinate system (b-frame) has its origin at the center of mass of the vehicle. Its axes are aligned with the vehicle’s pitch axis, roll axis, and yaw axis, pointing toward the right, forward, and upward directions of the vehicle’s motion, respectively.

### 2.2. Relationship Between Harmonic Error and Temperature

When operating in whole-angle mode, hemispherical resonator gyro-based inertial navigation systems (HRG-INS) often exhibit output errors containing high-order harmonic components, such as 4θ, 8θ, 12θ, 16θ-related errors. These errors introduce harmonic bias errors into the inertial navigation system. Moreover, the harmonic errors in the HRG output are highly sensitive to temperature. To investigate this relationship, multi-temperature tests were conducted. At each predefined temperature point, the HRG-INS was placed in a thermal chamber and soaked for over three hours to ensure a thermally stable internal state. Harmonic bias errors were then measured to analyze their temperature dependence.

**Figure 1:**
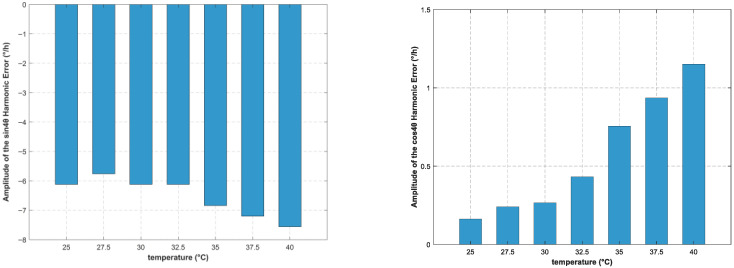
Temperature Dependence of Harmonic Bias Error for sin 4θ and cos 4θ.

As can be seen from the multi-temperature experimental results in Figure 1, the harmonic bias error is significantly influenced by temperature. Notably, the 4θ harmonic error exhibits the most pronounced temperature dependence. Higher-order harmonic errors such as 8θ, 12θ and 16θ have even smaller contributions to the overall bias error and can be effectively mitigated with appropriate compensation [28].

### 2.3. Relationship Between Temperature and Resonant Frequency

**Figure 2:**
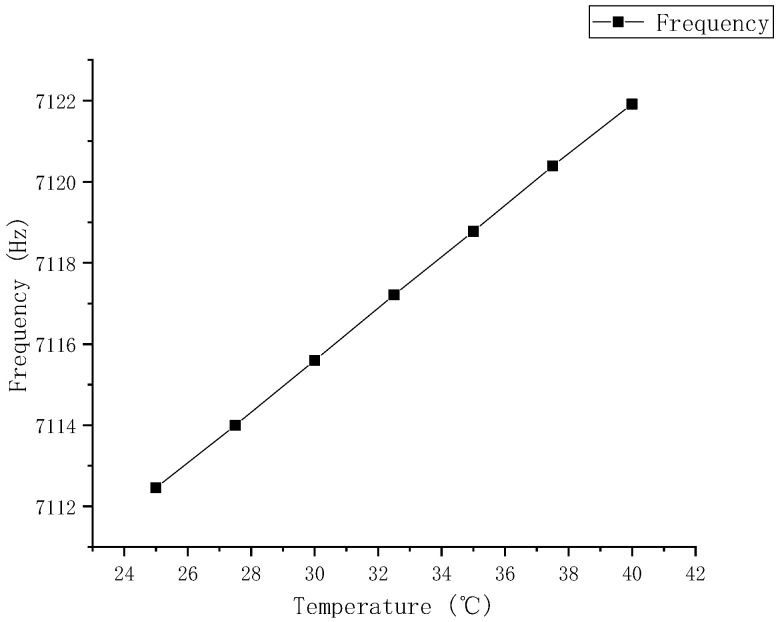
Relationship Between Temperature and Resonant Frequency.

Experimental results in Section 2.2 demonstrate that the output errors of the hemispherical resonator gyro, particularly the harmonic errors, are susceptible to temperature variations, making temperature compensation for these errors necessary. However, due to the vacuum environment inside the resonator, the internal temperature changes slowly and cannot be accurately measured. According to existing research, the resonant frequency of the hemispherical resonator gyro is influenced by temperature and exhibits an approximately linear relationship [29]. To verify the correlation between resonant frequency and temperature, we conducted relevant experiments and fitted the results, which indicate a linear relationship between temperature and resonant frequency as shown in Figure 2 (operating temperature range: 25–40 °C). Therefore, we will use the resonant frequency as a substitute variable for temperature in compensation.

### 2.4. Temperature (Frequency)-Dependent Harmonic Bias Error Model

**Figure 3:**
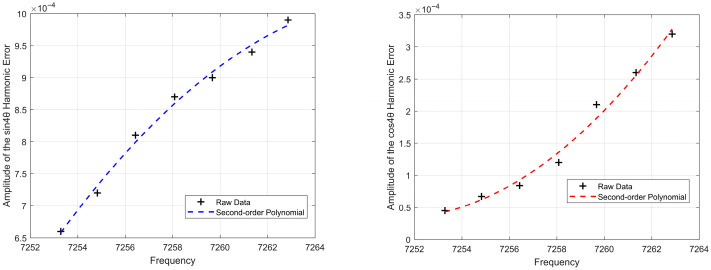
4θ Harmonic Bias Error Amplitude vs. Resonant Frequency.

A polynomial regression analysis was performed with the gyroscope’s resonant frequency as the independent variable and the amplitude of the 4θ harmonic bias error as the dependent variable. Based on comprehensive tests involving multiple temperature points, multiple gyroscopes, and repeated measurements, the results indicate that the optimal relationship between the amplitude of the 4θ harmonic bias error and the resonant frequency follows a quadratic function. Partial experimental results are shown in Figure 3.

Based on the experimental results, this study establishes a model of the bias harmonic error versus frequency as follows:

(2) $$\begin{array}{ll} \mathrm{ADB}\left(\theta ,f\right) & =\left(B_{s4}^{2}f^{2}+B_{s4}^{1}f+B_{s4}^{0}\right)\sin4\theta +\left(B_{c4}^{2}f^{2}+B_{c4}^{1}f+B_{c4}^{0}\right)\cos4\theta \\ & +B_{s8}\sin8\theta +B_{c8}\cos8\theta \\ & +B_{s12}\sin12\theta +B_{c12}\cos12\theta \\ & +B_{s16}\sin16\theta +B_{c16}\cos16\theta \end{array}$$

Here, $B_{\mathrm{sn}}$ denotes the amplitude of the sine component of the harmonic error, $B_{\mathrm{cn}}$ represents the amplitude of the cosine component of the harmonic error, with n taking values of 8, 12, and 16. $B_{s4}^{i}$ indicates the i-th order temperature (frequency) coefficient, where i ranges from 0 to 2.

The 4θ harmonic error is significantly influenced by temperature. Therefore, only the 4θ-related harmonic error is modeled as a temperature-dependent error. Other harmonic errors are less affected by temperature and can be approximated as constant values, which can be directly compensated through least squares fitting of the gyroscope’s output.

### 2.5. IMU Error Model

#### 2.5.1. Hemispherical Resonator Gyro Error Model

- Bias Error

Bias error refers to the output value of the hemispherical resonator gyro when the external angular velocity is zero, measured in °/h. It generally consists of two components: one is the constant bias, which can be compensated through calibration; the other is the gyro’s random walk noise, which is not compensatable. The mathematical model of the bias error is denoted as:

(3) $$\mathrm{gdrift}={\left[\begin{array}{ccc} B_{gcx} & B_{\mathrm{gcy}} & B_{\mathrm{gcz}} \end{array}\right]}^{T}+{\left[\begin{array}{ccc} B_{g4\theta x} & B_{g4\theta y} & B_{g4\theta z} \end{array}\right]}^{T}$$

(4) $$B_{g4\theta }=\left(B_{s4}^{2}f^{2}+B_{s4}^{1}f+B_{s4}^{0}\right)\sin4\theta +\left(B_{c4}^{2}f^{2}+B_{c4}^{1}f+B_{c4}^{0}\right)\cos4\theta$$

where θ is the precession angle of the gyro, f is the resonant frequency of the hemispherical resonator gyro, $B_{gc}$ denotes the conventional gyro bias error (excluding harmonic error terms), and $B_{g4\theta }$ represents the harmonic bias error.

- Scale Factor Error

The scale factor refers to the proportional relationship between the gyro output and the actual input angular velocity. Due to changes in the operating environment and prolonged operation time, the scale factor may vary. The deviation between the actual scale factor during operation and its initial calibrated value is referred to as the scale factor error, typically measured in parts per million (ppm). Its mathematical model is denoted as [30]:

(5) $${\tilde{K}}_{g}=K_{g}(I+\delta K_{g})$$

(6) $$\delta K_{g}=\left[\begin{array}{ccc} \delta K_{\mathrm{gx}} & 0 & 0\\ 0 & \delta K_{\mathrm{gy}} & 0\\ 0 & 0 & \delta K_{\mathrm{gz}} \end{array}\right]$$

where ${\tilde{K}}_{g}$ denotes the actual scale factor value, $K_{g}$ represents the initially calibrated scale factor value, and $\delta K_{g}$ corresponds to the gyro scale factor error.

- Installation Error

In an IMU, due to factors such as manufacturing and assembly deviations and structural deformation, there exists an error between the actual installation alignment of the gyroscopes and the ideal orthogonal coordinate system. By constraining and aligning the body frame with the gyro coordinate system, the installation errors can be reduced to three independent parameters.

(7) $$\delta M_{g}=\left[\begin{array}{ccc} 0 & 0 & 0\\ \delta M_{\mathrm{gyx}} & 0 & 0\\ \delta M_{\mathrm{gzx}} & \delta M_{\mathrm{gzy}} & 0 \end{array}\right]$$

where $\delta M_{\mathrm{gij}}$ (i=y, z; j=x, y; and i≠j) represents the installation error of the gyro between the i axis and the oij plane in the b-frame.

Based on the error definitions above, the output error model of the hemispherical resonator gyro can be expressed as follows:

(8) $$\delta \omega_{\mathrm{ib}}^{b}=(\delta K_{g}+\delta M_{g})\omega_{\mathrm{ib}}^{b}+B_{g}+\varepsilon_{g}$$

where $\omega_{\mathrm{ib}}^{b}$ is the angular velocity of frame b with respect to frame i, projected onto frame b, $\delta \omega_{\mathrm{ib}}^{b}$ represents the error in $\omega_{\mathrm{ib}}^{b}$, and $\varepsilon_{g}$ denotes the random bias of the gyro.

#### 2.5.2. Accelerometer Error Model

- Bias Error

Bias error refers to the output value of the accelerometer when the externally applied specific force is zero, typically measured in micro-g (μg). The bias generally consists of a constant component and a random component. The constant bias can be precisely calibrated and compensated, while the random bias is generally not compensatable. The mathematical model of the constant bias is denoted as:

(9) $$\mathrm{adrift}={\left[\begin{array}{ccc} B_{\mathrm{ax}} & B_{\mathrm{ay}} & B_{\mathrm{az}} \end{array}\right]}^{T}$$

- Scale Factor Error

The scale factor refers to the proportional relationship between the output pulses of the accelerometer and the actual input specific force. Due to changes in the operating environment and prolonged operation time, the scale factor may vary. The deviation between the actual scale factor during operation and its initial calibrated value is referred to as the scale factor error, typically measured in parts per million (ppm). Its mathematical model is denoted as [30]:

(10) $${\tilde{K}}_{a}=K_{a}(I+\delta K_{a})$$

(11) $$\delta K_{a}=\left[\begin{array}{ccc} \delta K_{\mathrm{ax}} & 0 & 0\\ 0 & \delta K_{\mathrm{ay}} & 0\\ 0 & 0 & \delta K_{\mathrm{az}} \end{array}\right]$$

where ${\tilde{K}}_{a}$ denotes the actual value of the scale factor, $K_{a}$ represents the initially calibrated value of the scale factor, and $\delta K_{a}$ corresponds to the scale factor error.

- Installation Error

During actual operation of the IMU, the installation error of the accelerometer is defined as the deviation between the actual installation parameters and their initially calibrated values, caused by factors such as structural deformation or changes in the calibration environment. This error is typically measured in arcseconds, and its mathematical model is expressed as [24]:

(12) $$\delta M_{a}=\left[\begin{array}{ccc} 0 & \delta M_{\mathrm{axy}} & \delta M_{\mathrm{axz}}\\ \delta M_{\mathrm{ayx}} & 0 & \delta M_{\mathrm{ayz}}\\ \delta M_{\mathrm{azx}} & \delta M_{\mathrm{azy}} & 0 \end{array}\right]$$

where $\delta M_{\mathrm{aij}}$ (i=y, z; j=x, y; and i≠j) represents the installation error of the accelerometer between the i axis and the oij plane in the b-frame.

Based on the error definitions above, the output error model of the accelerometer can be expressed as follows:

(13) $$\delta f^{b}=(K_{a}\delta K_{a}+\delta M_{a})N_{a}+B_{a}+\varepsilon_{a}$$

where $\delta f^{b}$ denotes the error in specific force, $N_{a}$ represents the number of output pulses from the accelerometer, and $\varepsilon_{a}$ indicates the random bias of the accelerometer.

### 2.6. Strapdown Inertial Navigation Error Equations

In practical inertial navigation systems, sensor measurement errors are inherent, and the initial alignment process also introduces certain deviations. These errors continuously propagate and accumulate through the navigation update equations. Therefore, the strapdown inertial navigation error equations form the basis for analyzing the error evolution in inertial navigation systems [31,32].

- Attitude Error Equations

(14) $${\dot{\phi }}^{n}=\phi^{n}\times \omega_{\mathrm{in}}^{n}+\delta \omega_{\mathrm{in}}^{n}-C_{b}^{n}\left(\left[\delta K_{g}\right]+\left[\delta M_{g}\right]\right)\omega_{\mathrm{ib}}^{b}-B_{g}^{n}$$

- Velocity Error Equations

(15) $$\begin{array}{l} \delta {\dot{V}}^{n}=-\phi^{n}\times f^{n}+C_{b}^{n}\left(\left[\delta K_{a}\right]+\left[\delta M_{a}\right]\right)f^{b}\\ +\delta V^{n}\times \left(2\omega_{\mathrm{ie}}^{n}+\omega_{\mathrm{en}}^{n}\right)+V^{n}\times \left(2\delta \omega_{\mathrm{ie}}^{n}+\delta \omega_{\mathrm{en}}^{n}\right)+B_{a}^{n} \end{array}$$

- Position Error Equations

(16) $$\begin{array}{l} \delta \dot{L}=\frac{\delta V_{N}}{R_{N}+h}-\delta h\frac{V_{N}}{{\left(R_{N}+h\right)}^{2}}\\ \delta \dot{\lambda }=\frac{\delta V_{E}}{R_{E}+h}\sec L+\delta L\frac{V_{E}}{R_{E}+h}\tan L\sec L-\delta h\frac{V_{E}\sec L}{{\left(R_{E}+h\right)}^{2}}\\ \delta \dot{h}=-\delta V_{D} \end{array}$$

This study incorporates the IMU error model established in Section 2.5 into the strapdown inertial navigation error equations, quantitatively deriving the variation patterns of navigation parameter errors (such as position, velocity, and attitude) caused by device errors of the hemispherical resonator gyro and accelerometer. This provides a theoretical foundation for the construction of a Kalman filter in system-level calibration.

## 3. System-Level Calibration Kalman Filter

System-level calibration aims to precisely estimate the bias harmonic error of the hemispherical resonator gyro at the inertial navigation system level. This paper designs a 48-dimensional Kalman filter capable of effectively calibrating various error parameters of the gyro—including the 4θ harmonic error and its temperature coefficients—as well as all error parameters of the accelerometer.

### 3.1. Construction of a 48-D Kalman Filter

State-Space Equation:

(17) $$\;\dot{X}=\mathrm{FX}+\mathrm{GW}$$

The state variable X:

(18) $$\begin{array}{l} X=\left[\begin{array}{ccccccccc} \phi_{N} & \phi_{E} & \phi_{D} & \delta V_{N} & \delta V_{E} & \delta V_{D} & \delta L & \delta \lambda & \delta h \end{array}\right.\\ \begin{array}{ccccccccc} B_{\mathrm{gx}} & B_{\mathrm{gy}} & B_{\mathrm{gz}} & B_{\mathrm{ax}} & B_{\mathrm{ay}} & B_{\mathrm{az}} & \delta K_{\mathrm{gx}} & \delta M_{\mathrm{gyx}} & \delta M_{\mathrm{gzx}} \end{array}\\ \;\begin{array}{cccccc} \delta K_{gy} & \delta M_{gzy} & \delta K_{gz} & \delta K_{ax} & \delta M_{ayx} & \delta M_{azx} \end{array}\\ \;\begin{array}{cccccc} \delta M_{\mathrm{axy}} & \delta K_{\mathrm{ay}} & \delta M_{\mathrm{azy}} & \delta M_{\mathrm{axz}} & \delta M_{\mathrm{ayz}} & \delta K_{\mathrm{az}} \end{array}\\ \;B_{\cos4\theta x}^{0}\;\;\;B_{\cos4\theta x}^{1}\;\;\;B_{\cos4\theta x}^{2}\;\;\;B_{\sin4\theta x}^{0}\;\;\;B_{\sin4\theta x}^{1}\;\;\;B_{\sin4\theta x}^{2}\\ \;B_{\cos4\theta y}^{0}\;\;\;B_{\cos4\theta y}^{1}\;\;\;B_{\cos4\theta y}^{2}\;\;\;B_{\sin4\theta y}^{0}\;\;\;B_{\sin4\theta y}^{1}\;\;\;B_{\sin4\theta y}^{2}\\ \;{\left.B_{\cos4\theta z}^{0}\;\;\;B_{\cos4\theta z}^{1}\;\;\;B_{\cos4\theta z}^{2}\;\;\;B_{\sin4\theta z}^{0}\;\;\;B_{\sin4\theta z}^{1}\;\;\;B_{\sin4\theta z}^{2}\right]}^{T} \end{array}$$

F is the state transition matrix of the system, and its specific form is determined by the attitude, velocity, and position error differential equations, namely, Equations (3)–(18).

(19) $$F={\left[\begin{array}{ccccccccccccc} F_{11} & F_{12} & F_{13} & F_{14} & 0_{3\times 3} & F_{16} & F_{17} & 0_{3\times 3} & 0_{3\times 3} & 0_{3\times 3} & F_{111} & F_{112} & F_{113}\\ F_{21} & F_{22} & F_{23} & 0_{3\times 3} & F_{25} & 0_{3\times 3} & 0_{3\times 3} & F_{28} & F_{29} & F_{210} & 0_{3\times 6} & 0_{3\times 6} & 0_{3\times 6}\\ 0_{3\times 3} & F_{32} & F_{33} & 0_{3\times 3} & 0_{3\times 3} & 0_{3\times 3} & 0_{3\times 3} & 0_{3\times 3} & 0_{3\times 3} & 0_{3\times 3} & 0_{3\times 6} & 0_{3\times 6} & 0_{3\times 6}\\ 0_{39\times 3} & 0_{39\times 3} & 0_{39\times 3} & 0_{39\times 3} & 0_{39\times 3} & 0_{39\times 3} & 0_{39\times 3} & 0_{39\times 3} & 0_{39\times 3} & 0_{39\times 3} & 0_{39\times 6} & 0_{39\times 6} & 0_{39\times 6} \end{array}\right]}_{48\times 48}$$

G is the noise distribution matrix, and W is the output noise of the gyros and accelerometer.

The observation equation can be expressed as:

(20) $$Z_{v,p}=H_{v,p}X+v_{v,p}$$

(21) $$H_{v,p}=\left[\begin{array}{cccc} 0_{3\times 3} & I_{3\times 3} & 0_{3\times 3} & 0_{3\times 39}\\ 0_{3\times 3} & 0_{3\times 3} & I_{3\times 3} & 0_{3\times 39} \end{array}\right]$$

where the velocity error and position error are taken as the observation vector $Z_{v,p}={\left[\begin{array}{cccccc} \delta V_{N} & \delta V_{E} & \delta V_{D} & \delta L & \delta \lambda & \delta h \end{array}\right]}^{T}$, $H_{v,p}$ is the observation matrix, and $v_{v,p}$ is the measurement noise.

### 3.2. Calibration Path and Observability

In system-level calibration, it is essential to design specific calibration paths for different error terms to fully excite all errors to be calibrated. This paper adopts a calibration path scheme comprising 19 distinct positions. Based on the Piece-Wise Constant System (PWCS) theory, the Structured Observability Matrix (SOM) is used instead of the Total Observability Matrix (TOM), and the rank of the SOM is computed for each position along the calibration path. It has been verified that in the designed system-level calibration scheme, the rank of the SOM equals the dimension of the system state variables, indicating that the system is fully observable; detailed information is provided in Table 1.

## 4. System-Level Calibration Experiments and Navigation Experiments

### 4.1. System-Level Calibration Experiments

**Figure 4:**
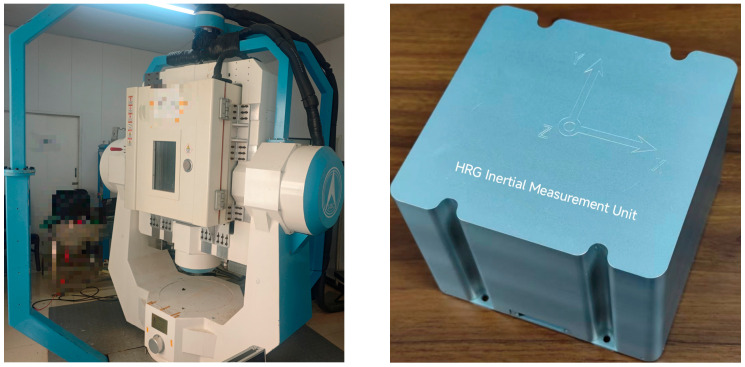
High-Precision Turntable and IMU.

The system-level calibration experiment was conducted by mounting the hemispherical resonator gyro IMU on a high-precision rotary table equipped with a temperature-controlled chamber, as illustrated in Figure 4. During the calibration process, programmed temperature variations were applied through the chamber to sufficiently excite the harmonic errors induced by temperature changes, thereby enabling precise identification of temperature-dependent error parameters.

**Figure 5:**
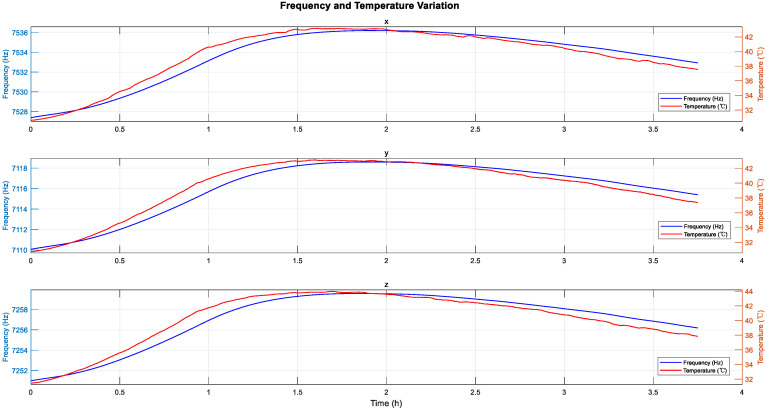
Frequency and Temperature Variations in the x, y, z Gyroscopes.

**Figure 6:**
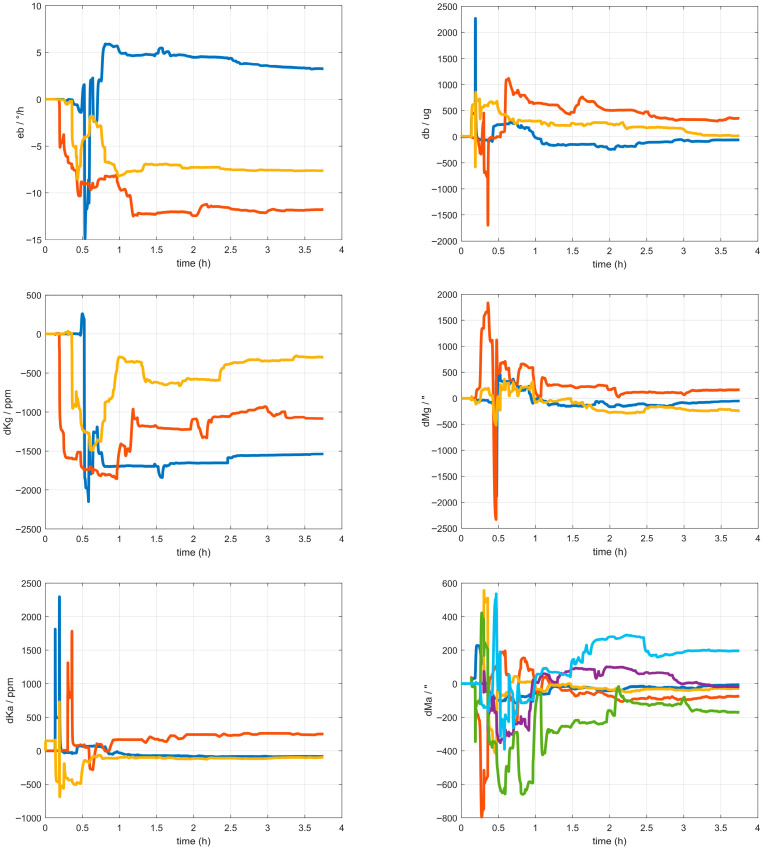

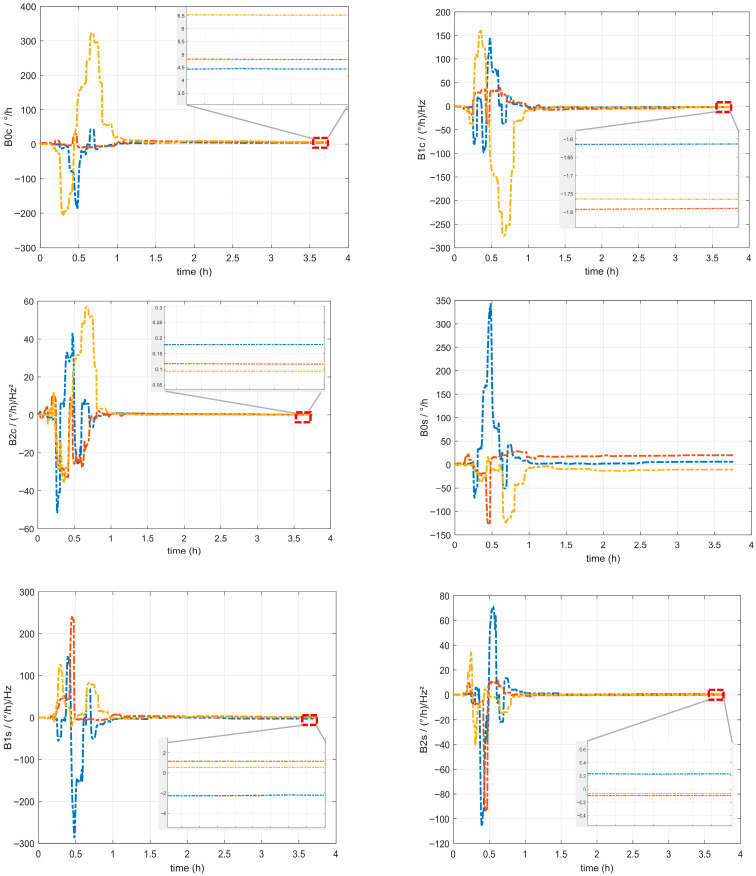
Convergence Curves of the Kalman Filter.

The hemispherical resonator gyro’s resonator operates in a vacuum environment. When external ambient temperature changes occur, the internal temperature of the gyro changes slowly, and the exact temperature cannot be determined. As illustrated in Figure 5, we utilize the oscillation frequency of the resonator as a substitute for the actual internal temperature of the gyro, which demonstrates a delayed response compared to the externally measured temperature. This is precisely why we have adopted frequency as a replacement for temperature in our compensation variable. The frequencies presented in Figure 6 represent the variations observed in three gyros during experiments when subjected to external temperature changes.

Calibration results are as follows:

In Figure 6, eb denotes the gyro bias, db represents the accelerometer bias, dKg($\delta K_{g}$) indicates the gyro scale factor error, dMg($\delta M_{g}$) refers to the gyro misalignment error, dKa($\delta K_{a}$) stands for the accelerometer scale factor error, and dMa($\delta M_{a}$) corresponds to the accelerometer misalignment error. Additionally, B0c and B0s symbolize the constant terms of the harmonic error’s cosine and sine components, respectively; B1c and B1s represent the first-order temperature (frequency) coefficients of the harmonic error’s cosine and sine components; and B2c and B2s denote the second-order temperature (frequency) coefficients of the harmonic error’s cosine and sine components, respectively. The red, blue, and yellow lines in the dMg plot represent the $\delta M_{\mathrm{gzx}}$, $\delta M_{\mathrm{gzy}}$ and $\delta M_{\mathrm{gyx}}$ components; in the dMa plot, the cyan, blue, purple, yellow, red, and green lines represent the $\delta M_{\mathrm{ayz}}$, $\delta M_{\mathrm{ayx}}$, $\delta M_{\mathrm{azy}}$, $\delta M_{\mathrm{axy}}$, $\delta M_{\mathrm{axz}}$ and $\delta M_{\mathrm{azx}}$ components; in the other figures, the blue, red, and yellow lines represent the error terms for the x, y, and z axes of the gyroscope or accelerometer.

From the experimental results of the system-level calibration (Figure 6 and Table 2), it can be observed that all state estimation curves of the Kalman filter exhibit favorable convergence characteristics. These results demonstrate that the proposed system-level calibration scheme effectively achieves precise identification and compensation of the bias harmonic errors and their temperature coefficients in the hemispherical resonator gyro-based inertial navigation system. This verifies the comprehensive performance of the proposed method in terms of the completeness of error modeling, the rationality of the filter design, and the effectiveness of the calibration process. The above outcomes fully confirm the feasibility and correctness of the proposed method, providing both a theoretical foundation and experimental support for its application in high-precision inertial navigation systems.

### 4.2. Navigation Experiments

This study designed a comparative navigation experiment involving three sets of IMUs for performance validation: the first set comprised a raw IMU (calibrated only at the individual component level) without system-level calibration or error compensation.; the second set was calibrated and compensated using a conventional 30-dimensional system-level calibration method; and the third set was processed using the proposed 48-dimensional system-level calibration approach.

Three hemispherical resonator gyro inertial navigation systems with different compensation schemes were mounted on a high-precision three-axis turntable to conduct navigation experiments under identical conditions. Each group followed the same rotation sequence and duration to simulate the actual rotational motion of the vehicle. The advantages and disadvantages of the three schemes were compared based on the experimental results.

**Figure 7:**
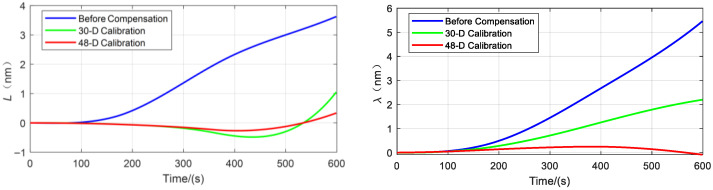
Comparison of Navigation Errors with Different IMU Compensation Methods.

**Figure 8:**
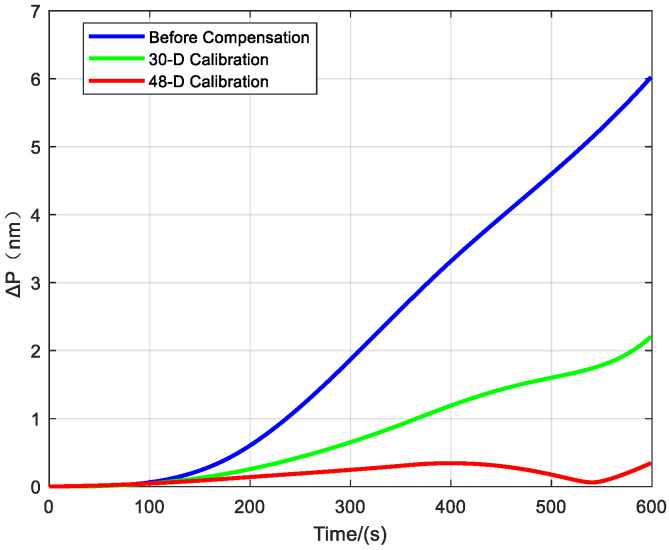
Navigation Radial Error.

The navigation experimental results are presented below:

Figure 7 shows the comparison of longitude, and latitude errors from the navigation experiment, while Figure 8 presents the comparison of radial position errors from the navigation experiment.

As shown in Table 3, the experimental results indicate that the navigation error of the scheme before compensation (with only individual calibration) is the largest, demonstrating that the errors of the hemispherical resonator gyro inertial navigation system are not fully compensated. The navigation accuracy of the 30-dimensional system-level calibration and compensation scheme is significantly improved compared to the individually calibrated scheme, with the maximum radial navigation error reduced from 6.02 nm to 2.2 nm. However, this scheme only compensates for the conventional inertial navigation system errors and does not suppress or compensate for the harmonic errors of the hemispherical resonator gyro. In contrast, the 48-dimensional system-level calibration and compensation scheme proposed in this paper achieves the highest navigation accuracy, effectively suppressing harmonic errors and reducing the maximum radial navigation error from 2.2 nm to 0.34 nm, thereby further enhancing navigation accuracy. Compared to the pre-compensation state, the maximum radial error is reduced by 94.35%. These experimental results demonstrate the superiority of the proposed method over existing approaches in error compensation for hemispherical resonator gyro inertial navigation systems.

## 5. Conclusions

This study investigates harmonic errors and their temperature dependence in hemispherical resonator gyro (HRG) inertial navigation systems through system-level calibration methodology. Experimental validation was first performed to confirm the correlation between harmonic errors and temperature variations, as well as the temperature-dependent characteristics of resonant frequency. Building on these findings, an enhanced HRG error model was developed, establishing a 48-dimensional state-space model that incorporates harmonic error terms and their temperature coefficients. A multi-position temperature-varying excitation trajectory was designed, with system observability analyzed using PWCS theory, enabling high-accuracy estimation of 4θ harmonic error coefficients. After implementing 48-dimensional system-level calibration and error compensation, navigation tests demonstrate remarkable improvements: the maximum radial error decreased from 6.02 nautical miles to 0.34 nautical miles (a 94.35% reduction), while the 50% circular error probability (CEP) improved from 1.86 nautical miles to 0.17 nautical miles (a 90.86% enhancement). Compared to single-gyro device-level calibration and discrete calibration approaches, the proposed method requires less time while achieving superior accuracy. When compared with 30-dimensional system-level calibration, this methodology enables calibration of HRG angle-dependent errors and effectively suppresses their impact. The approach presented in this paper significantly enhances the precision of hemispherical resonator gyro-based inertial navigation systems.

## Figures and Tables

**Table 1:** Rank of the Structured Observability Matrix (SOM).

Position	Rank	Position	Rank
1	12	11	48
2	18	12	48
3	24	13	48
4	30	14	48
5	36	15	48
6	39	16	48
7	42	17	48
8	45	18	48
9	47	19	48
10	48		

**Table 2:** System-Level Calibration Results (Kalman Filter Estimates).

Error Terms	X	Y	Z
eb (°/h)	3.25	−11.79	−7.62
db (μg)	−62.39	350.38	18.69
$\delta K_{g}$ (ppm)	−1538	−1083	−297
$\delta K_{a}$ (ppm)	−84.09	247.74	−97.24
$B_{0c}$ (°/h)	4.43	4.79	6.52
$B_{1c}$ ((°/h)/Hz)	−1.61	−1.79	−1.77
$B_{2c}$ ((°/h)/Hz^2^)	0.18	0.12	0.09
$B_{0s}$ (°/h)	5.93	19.54	−10.59
$B_{1s}$ ((°/h)/Hz)	−2.21	1.15	0.55
$B_{2s}$ ((°/h)/Hz^2^)	0.23	0.10	0.07
$\delta M_{g}(\prime \prime )$	$\delta M_{\mathrm{gyx}}$: −49.71	$\delta M_{\mathrm{gzx}}$: 160.14	$\delta M_{\mathrm{gzy}}$: −24.28
$\delta M_{a}(\prime \prime )$	$\delta M_{\mathrm{ayx}}$: −6.68	$\delta M_{\mathrm{azx}}$: −173.87	$\delta M_{\mathrm{axy}}$: −28.02
	$\delta M_{\mathrm{azy}}$: −17.63	$\delta M_{\mathrm{axz}}$: −69.96	$\delta M_{\mathrm{ayz}}$: 195.84

**Table 3:** Navigation Experimental Data under Different Compensation Schemes.

Schemes	Maximum Radial Error	50% CEP
Before Compensation	6.02 nm	1.86 nm
30-D	2.2 nm	0.65 nm
48-D	0.34 nm	0.17 nm

nm = nautical mile.

## Data Availability

Due to the sensitive nature of the data, we are unable to make them publicly available. However, reasonable requests for information regarding the data used in this study can be directed to the authors.
